# Radiographic Response Assessments and Standardized Imaging Interpretation Criteria in Head and Neck Cancer on FDG PET/CT: A Narrative Review

**DOI:** 10.3390/cancers16162900

**Published:** 2024-08-21

**Authors:** Jennifer A. Schroeder, Jorge D. Oldan, Valerie L. Jewells, Paul M. Bunch

**Affiliations:** 1 Department of Radiology, University of North Carolina School of Medicine, UNC Health, 101 Manning Drive, Chapel Hill, NC 27514, USA; 2 Department of Radiology, Wake Forest University School of Medicine, Atrium Health Wake Forest Baptist, Medical Center Drive, Winston Salem, NC 27157, USA; pbunch@wakehealth.edu

**Keywords:** MeSH, radiographic image interpretation, response evaluation criteria in solid tumors, squamous cell carcinoma of the head and neck, positron emission tomography-computed tomography, fluorodeoxyglucose F18, standardized interpretation criteria, positron emission tomography response criteria in solid tumors, Porceddu, Hopkins, NI-RADS, modified Deauville, Cuneo

## Abstract

**Simple Summary:**

Despite multiple standardized interpretation criteria (SICs) for imaging response assessment in patients with head and neck squamous cell carcinoma (HNSCC), these systems remain relatively underutilized. This underutilization may in part relate to the fact that the relevant information has been published in disparate journals over multiple decades. This paper aims to review the SICs available for use in interpreting post-treatment FDG PET/CT in patients with HNSCC. We evaluate each SIC in the context of eight desired traits. Selecting a SIC that best matches the needs of one’s practice is expected to facilitate multidisciplinary buy-in and maximize the likelihood of successful implementation.

**Abstract:**

Introduction: There is growing interest in the development and application of standardized imaging criteria (SIC), to minimize variability and improve the reproducibility of image interpretation in head and neck squamous cell carcinoma (HNSCC). Methods: “Squamous cell carcinoma” AND “standardized interpretation criteria” OR “radiographic response assessment” were searched using PubMed and Google Scholar for articles published between 2009 and 2024, returning 56 publications. After abstract review, 18 were selected for further evaluation, and 6 different SICs (i.e., PERCIST, Porceddu, Hopkins, NI-RADS, modified Deauville, and Cuneo) were included in this review. Each SIC is evaluated in the context of 8 desired traits of a standardized reporting system. Results: Two SICs have societal endorsements (i.e., PERCIST, NI-RADS); four can be used in the evaluation of locoregional and systemic disease (i.e., PERCIST, Hopkins, NI-RADS, Cuneo), and four have specific categories for equivocal imaging results (i.e., Porceddu, NI-RADS, modified Deauville, and Cuneo). All demonstrated areas for future improvement in the context of the 8 desired traits. Conclusion: Multiple SICs have been developed for and demonstrated value in HNSCC post-treatment imaging; however, these systems remain underutilized. Selecting an SIC with features that best match the needs of one’s practice is expected to maximize the likelihood of successful implementation.

## 1. Introduction

In the United States, over fifty-eight thousand new cases of head and neck squamous cell carcinoma (HNSCC) are diagnosed each year, and approximately twelve thousand patients died from this disease in 2023. The treatment options and prognosis vary but are affected by patient performance status and preferences, tumor site of origin (e.g., nasopharynx, sinus, oral cavity, oropharynx, larynx), and stage at diagnosis. Most HNSCC patients present with locoregional or distant metastases, which are associated with 5-year survival rates of 69% and 39%, respectively [1]. Given the anatomic complexities of HNSCC and the critical importance of accurate staging on management, these patients are best cared for by a multidisciplinary team of surgeons, oncologists, and radiation oncologists.

The central importance of diagnostic imaging in the initial evaluation of HNSCC is further underscored by the AJCC Cancer Staging eighth edition, which states that imaging assessment is essential for treatment selection. In addition, the value of diagnostic imaging for post-treatment surveillance is highlighted by the current NCCN guidelines, which recommend post-treatment imaging within 6 months for patients with T3 and T4 primary tumors and N2 or N3 nodal disease [2,3]. Other applications of diagnostic imaging in the context of HNSCC include the planning of radiation therapy and treatment response assessment [2,4]. Modalities commonly utilized in the assessment of these patients include contrast-enhanced computed tomography (CECT), fluorodeoxyglucose positron emission tomography (FDG PET/CT), and magnetic resonance imaging (MRI).

FDG PET/CT is particularly useful for initial staging, as it most accurately characterizes locoregional and distant sites of disease. FDG PET/CT is also very useful for post-treatment response assessment when performed at least 12 weeks after the completion of chemoradiation, as it is highly sensitive for persistent disease [5]. Furthermore, the presence (or absence) of residual FDG activity is prognostic for progression-free and overall survival [2,4,6,7]. A notable pitfall of FDG PET/CT is false positive results due to physiologic uptake along the aerodigestive tract. An additional pitfall is the inability to assess small disease foci including the cranial nerves. As such, combining FDG PET/CT with a diagnostic contrast-enhanced examination (e.g., CECT, MRI) is preferred to minimize false positive results and the potential for unnecessary biopsies and other interventions [8].

The increasing role of imaging in HNSCC highlights the need for effective communication between radiologists and referring physicians regarding imaging findings in the context of imaging reports. One of the major challenges associated with the traditional free-form (i.e., not standardized) reporting of imaging studies is the variability in how imaging findings are described by different imaging interpreters (e.g., radiologist, nuclear physicians, etc.). As a result, the perceived meaning of the report by a referring physician can substantially differ from the intended meaning [9,10,11]. In the context of FDG PET/CT for HNSCC, perceptions of disease status (i.e., complete response, partial response, progressive disease, indeterminate) vary among different oncology clinicians exposed to the same free-form report [12]. These same authors also observed minimal agreement between the oncology clinician perceived disease status and the response status assigned by the nuclear medicine physician in the FDG PET/CT report. This lack of agreement is particularly concerning, given that the nuclear medicine physician-assigned response status but not the oncology clinicians’ perceived status was significantly associated with overall survival [12]. 

In response to the challenges described above, there is growing interest in the development and application of standardized imaging criteria (SIC) as a tool for minimizing variability and improving the reproducibility of image interpretation in the context of HNSCC. The characteristics of SICs include the use of a lexicon for describing imaging findings and the use of an ordered numeric scale (e.g., 1–5) to communicate disease status. The benefits of SICs over free-form reporting include (1) increased efficiency in the act of interpretation, (2) decreased inter-reader variability, (3) decreased intra-reader variability, (4) enhanced communication between radiologists, (5) enhanced learning for trainees, (6) increased clarity of the clinical significance of the imaging findings, (7) improved risk stratification, and (8) improved data collection for clinical research to advance patient care [12,13,14,15]. Potential disadvantages include (1) time and effort associated with adopting a new reporting system, (2) limited flexibility when applied to complex cases, (3) and potential inconsistencies in the application of the SICs [13].

Although SICs offer many benefits and relatively few disadvantages, they remain underutilized in the context of HNSCC. One challenge when considering SIC adoption relates to the multiple available options, each with relative strengths and weaknesses. To better inform imagers and other members of the multidisciplinary team considering SIC adoption, we aim to provide a narrative review of SICs available for use in HNSCC that are applicable to FDG PET/CT and discuss if they are also applicable to CECT and MRI. This paper emphasizes the relative strengths, weaknesses, and desirable features of each SIC. We believe that by matching the features of the SIC to the needs of one’s practice, imagers can secure multidisciplinary buy-in and maximize the likelihood of successful implementation.

## 2. Search Strategy and Method

The terms “squamous cell carcinoma” AND “standardized interpretation criteria” OR “radiographic response assessment” were searched using PubMed and Google Scholar for articles published between 2009 and 2024, returning 56 publications. After reviewing the title and abstracts and methods of these results for relevance to response assessment in HNSCC, papers were excluded if FDG PET/CT was not an included imaging modality, the primary malignancy was not HNSCC originating from the aerodigestive tract, and/or if the methods did not clearly define the process of imaging response assessment in a way that was reproducible. As a result, 18 papers were selected for further evaluation.

These 18 papers described a total of 7 different SICs (PERCIST, Porceddu, Hopkins, NI-RADS, modified Deauville, Cuneo, and Choi) used for the post-treatment response evaluation of HNSCC. All 7 of these SICs have been applied to FDG PET/CT, and 1 (NI-RADS) has also been applied to CECT and MRI. One of these 7 SICs (i.e., the Choi criteria) is excluded from further discussion because the paper does not explicitly explain how this system, which was initially developed for gastrointestinal stromal tumors and incorporates lesional Hounsfield units, is to be applied to HNSCC [16]. The remaining 6 SICs are presented in this review.

To facilitate comparison of these 6 SICs available for use in HNSCC, we have characterized each SIC with respect to the following 8 desired traits, which are adapted from Pesapane et al.’s “10 Rules to Create a Standardized Report” [13]:

(1) Clarity and conciseness (ideally represented by a numerical scale);
(2) Incorporates relevant clinical information into the interpretation process;
(3) Uses consistent nomenclature (e.g., clear definitions or a lexicon);
(4) Provides a clinical response assessment (e.g., complete, partial, etc.);
(5) Uses structured reporting elements with a publicly available template;
(6) Incorporates imaging findings into recommendations for follow-up;
(7) Can be validated and facilitates peer review;
(8) Includes a mechanism for SIC updates and maintenance.

## 3. Results

The selected papers described an array of SIC evaluation methods. Most studies involved assigning and comparing SICs in a retrospective fashion; however, two original research studies implemented SICs prospectively [5,14]. Five of the papers were review articles [14,17,18,19,20], which either reviewed available SIC data as applied to radiographic response assessment, compared SIC qualitative or quantitative assessments, or compared different SICs to each other.

In the following sections, we discuss each SIC in detail based on the chronological order of introduction. We also provide a brief introduction to the Response Evaluation Criteria in Solid Tumors (RECIST) because the Positron Emission Tomography Response Criteria in Solid Tumors (PERCIST) is a derivative of RECIST. Similarly, we provide a brief discussion of the Deauville 5-point scale (Deauville 5PS) because, although this particular SIC was developed for lymphoma rather than HNSCC, several of the SICs used in HNSCC are derivatives of the Deauville 5PS. Finally, we summarize the most relevant information from our discussion into three tables.

Table 1 summarizes when the SICs were introduced and how (e.g., prospective study, retrospective study, other), endorsing organizations, imaging modalities to which the SICs can be applied, and the appropriateness of the SICs for assessing locoregional and/or systemic disease.

Table 2 characterizes each of the six SICs in relation to the eight desirable traits of interest.

Table 3 maps the response assessment categories from each SIC to what constitutes a negative finding, an equivocal finding, and a positive finding on the post-treatment imaging examination. A discussion of the various SIC, in order of introduction, follows.

**Figure 1:**
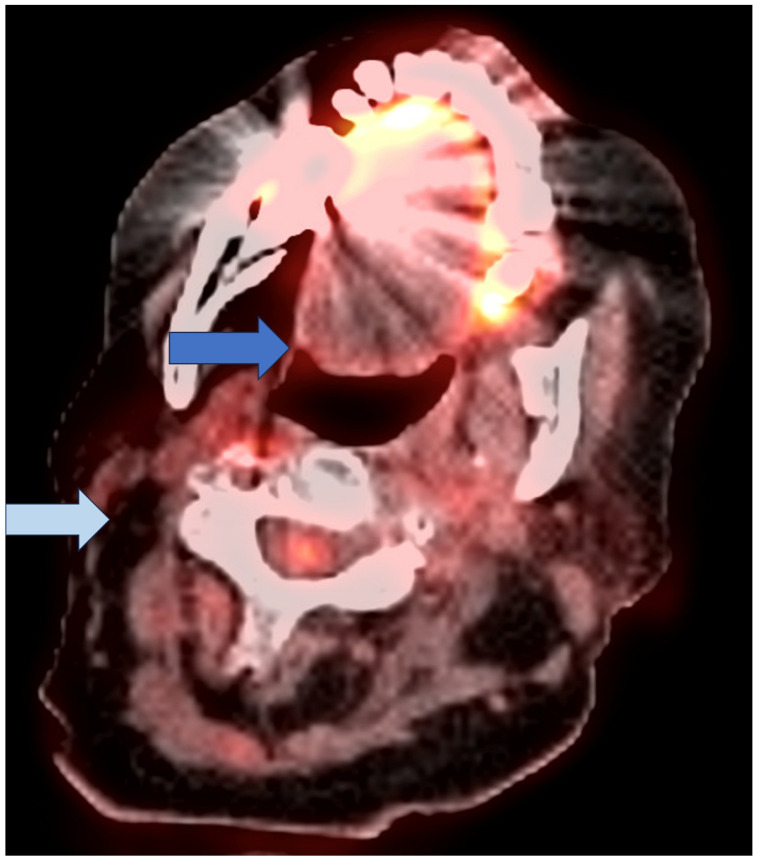
Axial fused image of a post-therapy FDG PET/CT in a 74-year-old woman status post-radiation of right tonsil cancer which demonstrates no residual FDG uptake in the right tonsil (dark blue arrow) nor in the previously avid nodes (all sites below mediastinal blood pool, marked by light blue arrow). This case represents a complete metabolic response by all criteria. More specifically, this exam would be assigned CR by PERCIST as the tumor has disappeared; H1 (negative) by Hopkins as the uptake is less than the IJV; N1 (no evidence of recurrence) by NIRADS as there is no uptake of visible mass on CT; M1 (negative) by MDS as the uptake is less than the mediastinal blood pool; C1 (negative) by Cuneo for the same reason; and P1 (negative) by Porceddu as there is no residual uptake above the mediastinal blood pool in the nodes.

**Figure 2:**
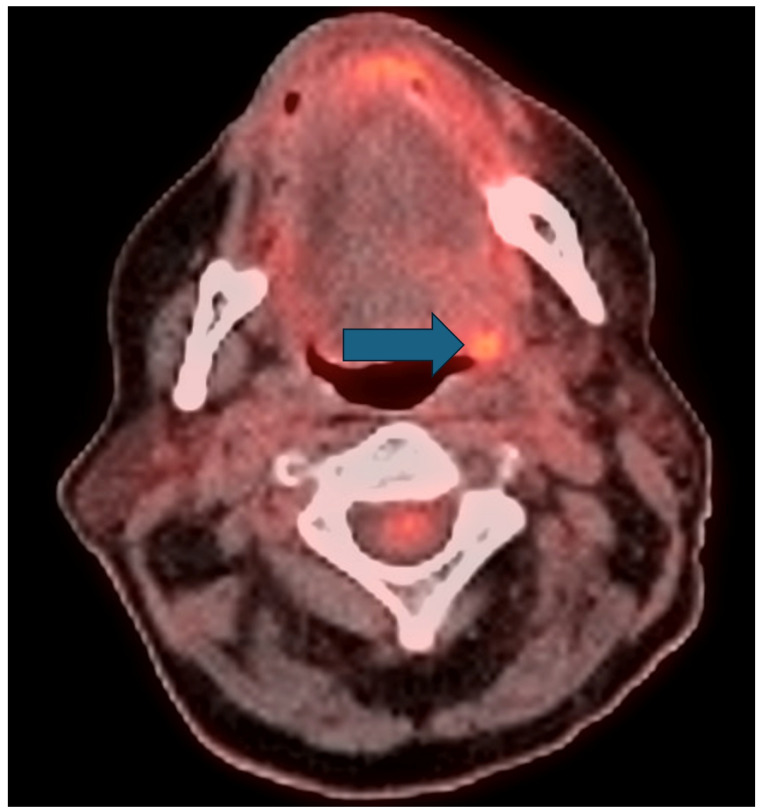
Axial fused image of a post-therapy FDG PET/CT in a 63-year-old woman with left tonsillar squamous cell carcinoma which demonstrates potentially ambiguous tongue uptake at the site of the primary tumor (uptake between that of the blood pool and the liver marked with arrow) that may result in a variable interpretation if written in free-form text. For this patient, this was the only residual site of uptake, thus determining her post-therapy disease status. Indeed, even by SICs there is variability in the resulting clinical assessment with both negative and equivocal disease assessments possible in this case. More specifically, this finding is assigned as PR by PERCIST given the large and incomplete decline in uptake; H2 (equivocal) by Hopkins, being between the IJV and the liver; NIRADS 2 (equivocal), having low-grade focal uptake; M2 (equivocal), being between the mediastinal blood pool and the liver; C 2 (negative) by Cuneo since the uptake is between the mediastinal blood pool and the liver but the background is less than the reference lesion. Porceddu does not evaluate the primary site and thus is not applicable for this exam.

**Figure 3:**
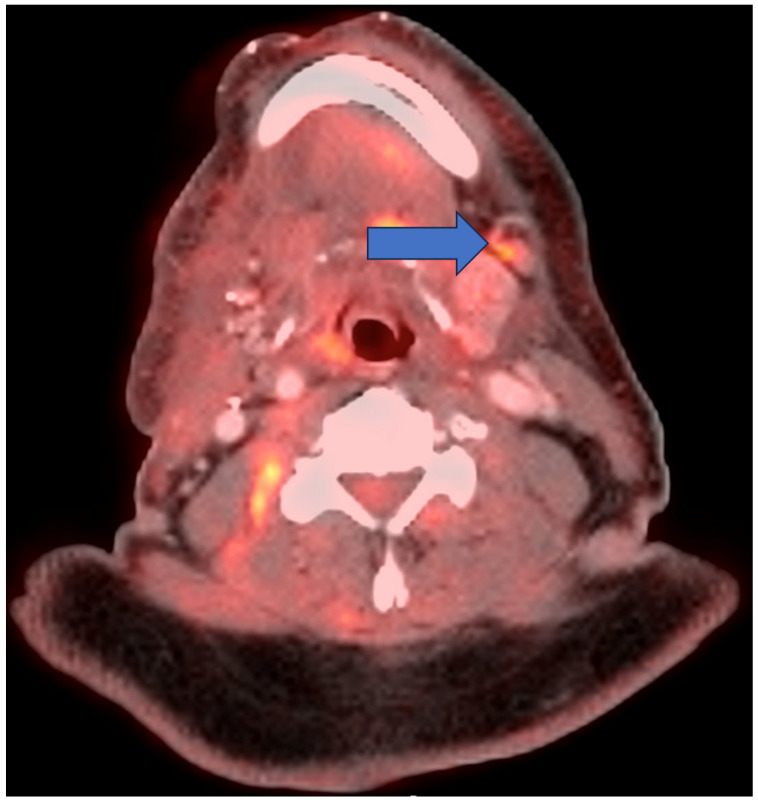
Axial fused image from a post-therapy FDG PET/CT in a 71-year-old woman with tongue cancer demonstrating a newly avid level 1B node (arrow) with uptake that is greater than in the liver and background tissues. By all criteria, this represents progressive disease. More specifically, this case is assigned PR by PERCIST given the incomplete decline; P3 (positive) by Porceddu given the uptake being greater than in the liver; H4 (positive) by Hopkins for the same reason; N3 (high suspicion) by NIRADS given the intense focal uptake; M4 (positive) by MDS for a new site of disease; and Cuneo 6 (positive) due to being greater than in the liver.

Some example imaging cases are represented in Figure 1, Figure 2 and Figure 3 as follows:

### 3.1. PERCIST

The very first imaging SIC that is applicable to HNSCC (RECIST) was developed in 1999 by the European Organization for Research and Treatment of Cancer (EORTC) in response to the need for a standardized imaging-based method of determining the treatment response across different solid tumor types. One decade later, PERCIST was proposed, which was also not specific to a particular type of cancer, but could be used in the evaluation of any malignancy imaged with FDG PET/CT. PERCIST was introduced by nuclear imagers to improve the consistency of interpretation of FDG PET/CT findings by using an objective scale to assess the lesional response to therapy in FDG avid disease states [17]. More specifically, PERCIST proposed four categories determined by the total change in the peak Standard Uptake Value (SUV): complete response, defined as the disappearance of all metabolically active tumors so that there is no residual uptake greater than the mean SUV of the liver; partial response, defined as a greater than 30% decrease in the peak SUV of pre-existing lesions in the absence of new sites of disease; progressive disease, defined as a greater than 30% increase in the SUV peak or confirmed new lesions; and stable disease, defined as persistent lesional activity that does not fit into the former categories (Table 3). A pooled analysis of RECIST and PERCIST performed in 2016 demonstrated the nearly identical performance of these two criteria, such that they could be used interchangeably [25]. In 2021, Kishikawa et al. showed that, although PERCIST and RECIST are associated with a similar negative predictive value, PERCIST is superior to RECIST with respect to the positive predictive value, further supporting FDG PET’s use in surveillance for the recurrence of FDG avid malignancies [26].

Considering the eight desired traits of a SIC, PERCIST fulfills four with the potential to fulfill three more, and lacks one (Table 2). More specifically, PERCIST has clear and concise language with four specific categories (i.e., complete response, partial response, stable disease, progressive disease) that could be assigned for the overall clinical assessment; however, a numerical scale—a feature of many other SICs that facilitates maximum conciseness—is not used [17]. While this SIC was developed to be applicable to all solid tumors—not just HNSCC—multiple studies have shown PERCIST’s value in HNSCC [26,27]. There have been recent updates to PERCIST, as therapies in solid tumors continue to change. For example, with the advent of immunomodulatory therapies, new types of treatment-related imaging response patterns have been identified (pseudoprogressive disease, hyper-progressive disease, and dissociated response). As such, the use of immunomodulating therapy is relevant information that needs to be accounted for when interpreting the imaging of these patients. Acknowledging these new patterns of disease, the European Association of Nuclear Medicine, the Society of Nuclear Medicine and Molecular Imaging, and the Australian and New Zealand Society of Nuclear Medicine published a joint statement in 2022 discussing modifications to PERCIST for use in patients being treated with immunomodulatory treatments [28]. An area for improvement for this SIC would be the development of a publicly available structured template for the implementation of PERCIST and its derivative criteria (such as imPERCIST) that would be accessible to interpreters of all backgrounds and practice types. Ideally, this template would be available on the endorsing societies’ websites. Lastly, PERCIST is lacking in one of the desired traits—the incorporation of imaging findings into recommendations for follow-up. In summary, PERCIST strictly fulfills four (clinical information, consistent nomenclature, validation/peer review, update/maintenance) of the eight desired SIC traits, has the potential to fulfill three (clear and concise—no numbers, structured reporting/template, provides clinical assessment), and lacks one (recommendations). Considering this is the first set of interpretation criteria created for FDG PET, PERCIST has many great attributes reflecting the forethought of its creators.

### 3.2. Deauville 5-Point Scale (Precursor to Porceddu, Hopkins, Modified Deauville Scale, and Cuneo)

In 2009, the same year that PERCIST was proposed, the now well-known Deauville 5PS was adopted for imaging response assessment in the reporting of FDG avid lymphomas. The Deauville 5PS’s approach to the FDG response criteria is novel and involves qualitatively comparing sites of disease to internal standards, specifically the patient’s mediastinal blood pool and liver on the same imaging examination. The Deauville 5PS uses these comparisons to categorize imaging findings into a concise and well-defined numerical five-point scale including the following: 1—no residual uptake; 2—uptake equal to or below the blood pool; 3—uptake above the blood pool but below or equal to the liver; 4—uptake slightly above the liver; 5—uptake greatly above the liver or any new lesion. It has since been shown that these FDG PET/CT-derived categories are significantly associated with clinical outcomes, which motivated the incorporation of these categories into the Lugano Classification in 2014 to translate the Deauville 5PS into a clinical response assessment (i.e., complete response, partial response, stable disease, progressive disease) [29]. As a result of the success of the Deauville 5PS in lymphoma, this approach has become the model for many subsequent FDG PET/CT SICs in other malignancies, including HNSCC. We discuss the following FDG PET/CT SICs derived from the Deauville 5PS in this review: Porceddu, Hopkins, modified Deauville scale, and Cuneo.

### 3.3. Porceddu Criteria

The first disease-specific SIC for FDG PET/CT to be used in the post-therapy imaging of patients with HNSCC was published by Porceddu et al. in 2011 [30]. This SIC was developed and applied in a prospective study aiming to better identify residual nodal disease among patients with a complete response at the primary site to target salvage neck dissection to those in need [Porceddu 2011] (Table 1). As such, this SIC is designed only to assess nodal disease. Analogous to the Deauville 5PS, the Porceddu criteria compare the nodal lesion FDG uptake to the liver with three possible categories: lesions more avid than in the liver (positive); lesions with an FDG uptake that is between ‘adjacent tissues’ and the liver (equivocal); and lesions with no residual uptake above background or diffuse uptake in the absence of a corresponding suspicious structural abnormality on anatomic imaging (negative) (Table 3) [30]. This SIC does not explicitly give numerical categories (e.g., 1–3), but its comparison to the liver is nevertheless concise, and it employs consistent nomenclature leading to a clinical assessment of the patient’s disease status with three resulting categories (residual/progressive disease, equivocal, and complete response) (Table 3). This SIC provides specific recommendations for clinical management based on FDG PET/CT findings that range from clinical observation for negative examinations (complete response), follow-up PET in 4–6 weeks for equivocal cases, and neck dissection in positive cases (residual/progressive disease). While its structure allows for peer review, there is no committee specifically tasked with updating and maintaining this SIC [14]. There is also no publicly available structured template, although the format of this criteria would be amenable to the development of one. Finally, this SIC does not incorporate clinical information (such as p16 positivity) into the imaging assessment. Of the eight desirable SIC traits, the Porceddu criteria fulfill four (provides clinical assessment, consistent nomenclature, recommendations, validation/peer review), has the potential to fulfill three (clear and concise—no numbers, structured reporting/template, and update/maintenance), and lacks one (use of clinical information) (Table 2).

### 3.4. Hopkins Criteria

In 2014, another SIC was introduced for the assessment of post-therapy FDG PET/CT in HNSCC patients in the form of the Hopkins criteria, which modified the Deauville 5PS for use in this disease state by comparing sites of suspected disease to the internal jugular vein (IJV) and liver [21]. The original study of this SIC was retrospective and looked at all sites of disease on post-therapy FDG PET/CT in HNSCC patients, giving individual scores for the primary, left neck, right neck, and overall assessment (Table 1). While this SIC does include the primary site in the evaluation, there are no special considerations or lexicons for use when evaluating the FDG findings at the primary mucosal site, even though the aerodigestive tract can have physiologic uptake approaching or even exceeding that of the IJV and liver. As a result, the potential for false-positive mucosal findings is relatively high with this SIC. The criteria scale is numerical and allows for five possibilities (1–5) with varying levels of suspicion and consistent nomenclature including the following: 1—uptake less than the IJV, representing a complete response; 2—focal uptake greater than IJV but less than in the liver, likely representing a complete response; 3—diffuse uptake greater than in the liver, likely representing post radiation inflammation and a complete response; 4—focal uptake at primary or nodes greater than in the liver, likely representing residual tumor and a partial response; and 5—focal and intense uptake much greater than in the liver, representing residual tumor and either a partial response or stable disease. A clinical assessment that is discussed in the original paper by Marcus et al. but not explicitly included in the Hopkins criteria scale is progressive disease, which is described as increasing uptake at prior sites of disease or new sites of uptake likely representing new metastatic disease (Table 3) [21]. The five numerical categories are clear (the IJV and liver are reference points, similar to Deauville) and concise; although, to be entirely consistent, there should be a numerical value assigned to progressive disease (PD), e.g., 6. The numerical assessment scores could easily be used as structured reporting elements, although no template is currently publicly available. The Hopkins criteria do not explicitly use clinical information in the imaging interpretation, but this SIC does offer clinical appraisal, as each numerical value is paired with a clinical response assessment. An important limitation of the Hopkins criteria is that this SIC does not include an indeterminate or equivocal category to capture patients that may benefit from closer clinical and/or imaging follow-up. Specific follow-up recommendations are not given for scans/patients who fall into each assessment category. Currently, there is no formal process for updating the Hopkins criteria, but this SIC has been used and validated in multiple subsequent studies [14,31,32,33]. Of the eight desirable traits, the Hopkins criteria fit two criteria (validation, consistent nomenclature), have the potential to fit four (clear and concise—no numerical rating for progressive disease, structured reporting/template, provides clinical assessment, update/maintenance), and are lacking in two (use of clinical information, recommendations) (Table 2). This is likely due to the age of the criteria, as they represent one of the earlier sets of criteria to be developed. A committee could easily bring it up to date, given the literature that is now available.

### 3.5. NI-RADS

Modeled after BI-RADS for breast imaging, the Neck Imaging and Reporting and Data System (NI-RADS) was initially introduced in 2016 by Aiken et al. [22]. Subsequently, an ACR-endorsed NI-RADS paradigm was published in 2018 [34]. A unique feature of this SIC is that it can be applied to CECT and MRI in addition to FDG PET/CT. Another distinctive trait of this SIC is that, in addition to the ACR endorsement, an ACR committee was formed in 2016 that is tasked with maintaining and updating NI-RADS with respect to the available literature. This SIC includes separate assessments of the primary site and the neck and uses separate and very specific lexicons for the reporting of each site (Table 1). This SIC has five categories, each with a corresponding management recommendation: 0—incomplete, assign a score in an addendum once relevant prior imaging examinations are available; 1—no evidence of recurrence, routine surveillance; 2—low suspicion of recurrence, direct visual inspection (for mucosal abnormalities) or short-interval follow-up imaging (for deep abnormalities); 3—high suspicion of recurrence, biopsy; 4—definitive recurrence, clinical management. Separate scores are assigned to the primary site and to the neck. Categories 1 through 4 have been shown to risk-stratify patients with respect to the likelihood of recurrence in multiple studies assessing multiple different imaging modalities (i.e., FDG PET/CT, CECT, MRI) [27,35,36,37,38,39,40]. This SIC can also be used in the post-therapy imaging assessment of head and neck cancers other than HNSCC, such as primary salivary malignancies. In addition to the linked management recommendations for each NI-RADS numerical category, the ACR NI-RADS Committee has also published an atlas with accompanying modality-specific lexicons [41]. A caveat to the provided lexicons is that some of the imaging descriptors are qualitative, rather than quantitative, which may result in inter-reader variability. To help with the implementation of this SIC, there are modality-specific structured reporting templates available on the ACR’s website [23,24]. Furthermore, it is possible to adapt the NI-RADS framework to meet the needs of one’s clinical practice and achieve stakeholder buy-in, which can increase the likelihood of successful implementation [42,43]. The only desired trait that the NI-RADS does not fulfill, is to systematically ‘incorporate relevant clinical information into the interpretation;’ although, the ACR does recommend that the interpreting imager review all the available clinical data before rendering an interpretation. In summary, NI-RADS fulfills seven traits (clear and concise, structured reporting/template, clinical assessment, consistent nomenclature, recommendations, validation/peer review, update/maintenance) and does not fulfill one (use of clinical information) (Table 2).

### 3.6. Modified Deauville Scale (MDS)

Sjovall et al. first used the pre-existing Deauville criteria to evaluate HNSCC in 2016 [18]. These authors used the mediastinal blood pool and liver in roughly the same fashion as the original Deauville 5PS, with assessment categories including the following: 1—no uptake, representing a complete metabolic response; 2—uptake less than mediastinum, probably representing a complete metabolic response; 3—uptake greater than the mediastinum but less than or equal to the liver, probably representing post-radiation inflammation; 4—uptake moderately increased compared to the liver, probably representing a persistent tumor; and 5—markedly increased uptake compared to the liver, representing residual or progressive disease. The cut-points are similar to the original Deauville 5PS except that in the context of lymphoma, a two is counted as negative and a four as a partial response.

The Deauville 5PS was further modified by Zhong et al. to a four-point scale (i.e., the modified Deauville scale—MDS) for harmonization and use in their 2020 study (Table 1) [14]. The MDS assessment categories include the following: 1—no uptake above the mediastinal blood pool, representing a complete response; 2—uptake between the mediastinal blood pool and the liver, indeterminate for residual disease; 3—uptake that has decreased but remains greater than in the liver at a site of prior disease, representing a partial response; and 4—new sites of uptake, representing progressive disease (Table 3). These imaging-based categories are concise and clear because this SIC uses the mediastinal blood pool and liver as internal reference points, thus providing consistent nomenclature which also maps to a clinical assessment of the imaging findings. Together, these three traits allow for ongoing peer review. This SIC is easily adopted by any nuclear imaging interpreter who has experience in reading PET for FDG avid lymphoma. The relatively brief descriptions could be used as structured reporting elements, although no template is currently publicly available. Like many of the other SICs, the MDS would be amenable to updates, though there is no organized group currently charged with this task. In summary, the MDS fulfills four (clear and concise, provides clinical assessment, consistent nomenclature, validation/peer review), has the potential to fulfill two (structured reporting/template, update/maintenance), and does not fulfill two (use of clinical information, recommendations) of the SIC desired traits (Table 2).

### 3.7. Cuneo Score

The Cuneo score, first described in 2020 by Bonomo et al., is an additional SIC created for use in the interpretation of post-therapy FDG PET/CT in patients with HNSCC. This SIC is intended for the assessment of both locoregional and systemic disease. Like the MDS, the Cuneo score involves making internal comparisons to the mediastinal blood pool and liver, but an important difference is that the Cuneo score expands the number of diagnostic categories to six by taking into consideration the local background. The creators of this SIC assert that incorporating the local background into the assessment improves specificity by excluding cases with a high background inflammatory uptake that would otherwise (often erroneously) be labeled as residual disease merely because the local uptake was greater than in the liver. The scale is as follows: 1—no uptake above the mediastinal blood pool, representing a complete response; 2—uptake between the mediastinal blood pool and the liver with absent local background activity or local background activity less than the reference lesion, probably representing a complete response; 3—residual uptake between the mediastinal blood pool and the liver with local background activity greater than the reference lesion, representing an equivocal result; 4—focal uptake slightly greater than in the liver with local background less than the reference lesion, representing an equivocal result; 5—focal uptake slightly greater than in the liver with local background greater than the reference lesion, likely representing residual disease; and 6—focal uptake more intense than in the liver, representing residual disease (Table 3). The language used to assign the imaging categories is somewhat concise as it gives a numerical scale from one to six. These six categories can be mapped to three clinical assessments with one and two as negative, three and four as equivocal, and five and six as positive [19,44]. This SIC has the potential for a structured report template (though none is publicly available) and could be easily peer-reviewed; however, this SIC has not yet been externally validated. Currently, there is also no organized group tasked with maintaining or updating this SIC, and relevant clinical information is not incorporated into the interpretation process. Finally, there are no accompanying recommendations for clinical management. Of the eight desirable traits, the Cuneo score fulfills three (clear and concise, provides clinical assessment, validation/peer review), has the potential to fulfill three (structured reporting/template, consistent nomenclature, update/maintenance), and does not fulfill two (use of clinical information, recommendations) (Table 2).

## 4. Discussion and Direct SIC Comparisons

Most SICs utilize consistent nomenclature with the more recently developed NI-RADS, Hopkins, MDS, and Cuneo score, all with strict numerical scales, which optimize conciseness and clarity in reporting. Each SIC provides a correlating clinical assessment for each imaging assessment category. However, some nomenclature systems fail to capture the full breadth of possible clinical responses. For instance, the Hopkins criteria have no category for progressive disease. Another example is the representation of patients with equivocal findings. Four of the six SICs (Porceddu, NI-RADS, MDS, and Cuneo score) have imaging assessment categories that map to an equivocal category, thus identifying a group of patients who are most likely benefit from short interval imaging or clinical follow-up and allow for further diseases risk stratification (Figure 2).

As expected, all six SICs are structured such that they can be, and many have been evaluated in the context of original research. Although, there are different levels of validation, applicability, and clinical utility with different SICs. For instance, only some versions of PERCIST (e.g., imPERCIST) incorporate relevant clinical data in the interpretation considerations, although NI-RADS suggests reviewing clinical data at the time of imaging interpretation [28]. Collaboration between radiologists, oncologists and radiation oncologists to formulate a method of incorporation of clinical data for NI-RADS (i.e., follow-up imaging, biopsy, and neck dissection) by the ACR committee would be a fruitful endeavor for this. Indeed, only PERCIST and NI-RADS have an established mechanism for updates and maintenance to incorporate the most recent available data, which is supported and organized by their endorsing societies (SNMMI and ACR, respectively).

There is variable inclusion of the primary site of disease into the clinical assessment with only Hopkins and NI-RADS allowing for separate and sometimes individualized primary site assessments. Only two SIC (Porceddu and NI-RADS) provide explicit recommendations for clinical management (e.g., follow-up imaging, biopsy, neck dissection) based on imaging findings. Only NI-RADS can be applied to imaging modalities other than FDG PET/CT and to histologies other than SCC. Additionally, only NI-RADS offers modality-specific reporting templates on a publicly available website (cite ACR site). None of these SIC dictate suggestions regarding chemotherapy or radiation therapy and instead leave these decisions up to the clinical care team.

The most detailed work directly comparing SIC relevant to the interpretation of post-therapy HNSCC imaging has been performed by Zhong et al., who evaluated NI-RADs, Porceddu, Hopkins, and the MDS after harmonizing each into a four-point scale to determine sensitivity, specificity, positive predictive value, negative predictive value, and accuracy in a cohort of 562 patients [14]. The authors found that all of the SIC in the study had high specificity, positive predictive value, and negative predictive value with similar accuracy for predicting clinical outcome. However, the number of equivocal results varied widely among the SIC, with the Hopkins criteria having the fewest and NI-RADS having the most [14]. The impact of whether this is a positive or a negative regarding outcomes needs further study. The lack of determinate results, i.e., the assignment of an equivocal assessment, may result in closer clinical follow-up with increased cost, but may also result in disease progression being caught at an earlier time point.

One interesting finding of the Zhong et al. study is that the negative predictive value of all SIC was higher in patients with human papilloma virus-associated (HPV+) cancers than in non-HPV-associated (HPV–) cancers. This means that while most patients with initial equivocal post-treatment FDG PET/CT at 12 weeks went on to meet criteria for complete response by short interval follow-up FDG PET/CT conducted before 30 weeks, HPV+ patients were more likely than HPV– patients to have recurrence on imaging by 30 weeks. This is despite the fact that HPV+ patients tend to have a better response to treatment in general [45]. Zhong et al. therefore concluded that patients with HPV+ cancers and equivocal initial post-treatment imaging results should be assigned to short interval follow-up with PET/CT or CECT (if PET/CT is not an option) in 4–6 weeks [14]. These HPV-related findings are an example of how comparative studies can further improve SIC by elucidating clinical factors that might be useful for improving imaging-based risk stratification.

Bonomo et al. retrospectively compared the Hopkins Criteria to the Cuneo Scale and the modified Deauville scale [44]. In the blinded review of post treatment PETs for HNSCC, those exams labeled as positive by the Cuneo score had the highest positive predictive value for residual disease, with patients scoring five or six on the Cuneo scale having a significantly worse prognosis. The Cuneo Score also demonstrated the highest accuracy among Cuneo, Deauville, and Hopkins [44]. A similar study comparing these SIC to NIRADS may be appropriate. In a separate study, the Hopkins criteria was found to have a 92% negative predictive value for residual nodes [46]. The high positive predictive value of the Cuneo score in irradiated lymph nodes likely relates to the fact that only this SIC considers the local background FDG activity in the cancer risk assessment, thereby decreasing false positives related to post-treatment inflammation of surrounding tissues [20].

Finally, NI-RADS is the only SIC that can be applied to CECT and MRI in addition to FDG PET/CT [47,48]. This capability for use across multiple modalities may be advantages in several scenarios such as when the clinical team chooses to alternate between surveillance anatomic (i.e., CECT or MRI) and metabolic (i.e., FDG PET/CT) imaging. Another is when FDG PET/CT is used to risk-stratify equivocal CECT or MRI surveillance imaging studies. Finally, there are some situations where the risk for false negative FDG PET/CT is increased and in which MRI has an advantage, such as skull base imaging to assess for intracranial extension and perineural tumor spread [49,50].

In short summary, two of the SIC have societal endorsements which also relegate committees to maintain the validity of the criteria in the context of recent literature (i.e., PERCIST, NI-RADS); four can be used in the evaluation of locoregional and systemic disease (i.e., PERCIST, Hopkins, NI-RADS, Cuneo), and four have specific categories for equivocal imaging results (i.e., Porceddu, NI-RADS, Modified Deauville, and Cuneo). All SIC demonstrated areas for future improvement in the context of the eight desired traits with Hopkins demonstrating the greatest room for growth and NI-RADS with the least.

## 5. Conclusions

In conclusion, SIC use offers advantages over traditional free-form reporting, particularly regarding effective multidisciplinary communication. Even though multiple SICs have been developed for and demonstrated value in the post-treatment imaging of patients with HNSCC, these systems remain relatively underutilized. In our experience, SIC underutilization largely relates to the time and effort required to choose and adopt one of the available options as well as uncertainty regarding the specific benefits and limitations of each SIC. To better inform radiologists and other members of the multidisciplinary head and neck cancer team considering SIC adoption, we have summarized the methodology of and literature supporting PERCIST, the Porceddu criteria, the Hopkins criteria, NI-RADS, the MDS, and the Cuneo score in the context of eight desired traits of the optimal SIC. Selecting an SIC with features that best match the needs of one’s practice and a willingness to adapt the selected SIC when necessary are likely to aid multidisciplinary buy-in and maximize the likelihood of successful implementation.

## Figures and Tables

**Table 1:** Summary of the available SICs for HNSCC.

	PERCIST Criteria	Porceddu	Hopkins Criteria	NI-RADS	Modified Deauville Scale	Cuneo Score
***Introduced***	2009 [17]	2011 [5]	2014 [21]	2016 [22]	2020 [14,22]	2020 [19]
***Development Methodology***	Review of existing criteria; proposed PERCIST based on literature review and expert opinion	Prospective original research	Retrospective original research	Multidisciplinary single institution development and implementation	Prospective original research Preceded by Deauville 5PS (2009) for FDG avid lymphoma and by first application of Deauville 5PS to HNSCC in 2016	Multicenter retrospective original research
***Applicable Cancers***	Any (i.e., not specific to HNSCC)	HNSCC	HNSCC	Any primary malignancy of the head and neck (HNSCC or other)	HNSCC	HNSCC
***Applicable Modalities***	FDG PET	FDG PET	FDG PET	FDG PET, CECT, MRI	FDG PET	FDG PET
***Site(s) of Disease Assessed***	Locoregional and systemic Does not differentiate between primary site and nodal disease	Nodal only	Locoregional and systemic Separate assessments for primary site, left neck, right neck, and overall.	Locoregional and systemic Separate assessments for primary site and nodal disease.	Nodal and systemic No primary site assessment	Locoregional and systemic
***Society Endorsements***	RSNA EANM SNMMI ANZSNM	None	None	ACR	None	None

Abbreviations: ACR—American College of Radiology; ANZSNM—Australian and New Zealand Society of Nuclear Medicine; Deauville 5PS—5-Point Deauville Scale; EANM—European Association of Nuclear Medicine; HNSCC—head and neck squamous cell carcinoma; NI-RADS—Neck Imaging Reporting and Data System; PERCIST—Positron Emission Tomography Response Criteria in Solid Tumors; SIC—standardized interpretation criteria; SNMMI—Society of Nuclear Medicine and Molecular Imaging; RSNA—Radiological Society of North America.

**Table 2:** Summary of the 6 SICs in relation to the adapted 8 desirable traits of effective standardized reports [13].

*Desired SIC Traits*	PERCIST	Porceddu	Hopkins Criteria	NI-RADS	Modified Deauville Scale	Cuneo Score
*Clear and concise (ideally represented by a numerical scale)*	Potential Defines 4 response categories (CR, PR, PD, and SD) but does not use a numerical scale	Potential Defines 3 response categories (negative, equivocal, residual/recurrent disease) but does not use a numerical scale	Potential Defines 5 numerical categories (1–5) that map to clinical response assessment but does not include a numerical category for PD	Yes Defines 5 numerical categories (0–4) that map to a level of suspicion for recurrent malignancy	Yes Defines 4 numerical categories (1–4) that map to clinical response assessments	Yes Defines 6 numerical categories (1–6) that map to clinical response assessments
*Incorporates relevant clinical information into the interpretation process*	Yes Clinical information (e.g., patient receiving immunotherapy) can impact how imaging assessment is performed in some versions of PERCIST (e.g., iPERCIST)	No	No	No Clinical information does not explicitly impact imaging assessment; however, this SIC recommends the interpreter review available clinical data prior to assessment	No	No
*Uses consistent nomenclature (e.g., clear definitions or a lexicon)*	Yes	Yes	Potential 5-point lexicon also uses qualitative assessments (focal/diffuse) that are not clearly defined and may lead to inter-reader variability	Yes	Yes	Potential 6-point lexicon uses qualitative assessments (focal/diffuse) that are not clearly defined and may lead to inter-reader variability
*Provides clinical response assessment and includes a category for equivocal imaging findings*	Potential Includes categories mapped to the overall clinical response assessment (CR, PR, PD, SD) but does not allow for equivocal imaging findings in all versions	Yes	Potential Numerical categories (1–5) map to clinical response assessments but do not allow for equivocal imaging findings	Yes	Yes	Yes
*Uses structured reporting elements with a publicly available template*	Potential Although none currently available	Potential Although none currently available	Potential Although none currently available	Yes Modality-specific templates available on the ACR website [23,24]	Potential Although none currently available	Potential Although none currently available
*Incorporates imaging findings into recommendations for clinical management*	No	Yes	No	Yes	No	No
*Can be validated and facilitates peer review*	Yes	Yes	Yes	Yes	Yes	Yes
*Includes mechanism for SIC updates and maintenance*	Yes	Potential No organized group currently charged with this task	Potential No organized group currently charged with this task	Yes	Potential No organized group currently charged with this task	Potential No organized group currently charged with this task
*Sum of desirable SIC traits*	Yes—4 Potential—3 No—1	Yes—4 Potential—3 No—1	Yes—1 Potential—5 No—2	Yes—7 Potential—0 No—1	Yes—4 Potential—2 No—2	Yes—3 Potential—3 No—2

Abbreviations: CR—complete response; PR—partial response; SD—stable disease; PD—progressive disease; MBP—mediastinal blood pool.

**Table 3:** Summary of SIC response assessment categories mapped to what constitutes negative, equivocal, and positive imaging findings.

Clinical Assessment for Residual Tumor	Negative	Equivocal	Positive	Other
CRITERIA				
PERCIST: Clinical assessment (CR, PR, PD, SD) assigned based on lesional FDG accumulation compared to baseline.	CR—disappearance of all metabolically active tumors so that there is no residual uptake greater than the mean SUV of the liver		PR—>30% decline in SUV peak between lesions before and after treatment PD—>30% increase in SUV peak or confirmed new lesions. SD—persistent lesional activity that does not fit CR, PR, or PD.	
Porceddu: 1–3	P1—Visual assessment demonstrating no residual uptake above background or diffuse uptake in the absence of a corresponding suspicious structural abnormality	P2—Visual assessment demonstrating focal uptake greater than adjacent normal tissues but below background liver activity	P3—Visual assessment demonstrating focal uptake corresponding to a structural abnormality of greater intensity than in the liver	
Hopkins: 1–5 and other/PD	H1—uptake less than internal jugular vein (IJV), CR H2—focal uptake greater than IJV but less than in the liver, likely CR H3—diffuse uptake greater than in the liver, likely post radiation inflammation and CR		H4—focal uptake at primary or nodes greater than in the liver, likely residual tumor PR H5—focal and intense uptake much greater than in the liver, representing residual tumor, either PR or SD Other—not included in the numeric scale but includes patients with PD (a new lesion that was not present at baseline)	
NI-RADS: N0–4	N1—no evidence of recurrence	N2—low suspicion for recurrence	N3—high suspicion for recurrence N4—definitive recurrence	N0— incomplete
Modified Deauville Score: M1–4	M1—No uptake above MBP, CR	M2—uptake between MBP and liver, indeterminate for residual disease	M3—uptake that has decreased from pretherapy imaging but remains greater than in the liver at a site of prior disease M4—new focal uptake, PD	
Cuneo Score C1–6	C1—no residual uptake above MBP, CR C2—focal uptake greater than MBP but less than in the liver with absent local background activity or local background activity less than the reference lesion, probably CR	C3—uptake greater than MBP but less than in the liver with local background activity greater than the reference lesion, indeterminate C4—focal uptake slightly greater than in the liver with local background less than the reference lesion, indeterminate	C5—focal uptake slightly greater than in the liver with local background greater than the reference lesion, likely residual disease C6—focal uptake greater than in the liver, residual disease	

Abbreviations: P—Porceddu; H—Hopkins; N—NI-RADS; M—modified Deauville score; C—Cuneo Score. IJV—internal jugular vein, MBP—mediastinal blood pool. CR—complete response, PR—partial response, PD—progressive disease, SD—stable disease.

## Data Availability

No new data were generated, only information abstracted from literature review. Summarized results are included in the tables within this review.
